# Management of acute disturbance: The intravenous route

**DOI:** 10.1192/j.eurpsy.2021.977

**Published:** 2021-08-13

**Authors:** M. Castro, M. Butler, A. Thompson, S. Gee, S. Posporelis

**Affiliations:** 1 Psychosis Studies, Institute of Psychiatry, Psychology and Neuroscience, London, United Kingdom; 2 Psychological Medicine, South London and Maudsley NHS Foundation Trust, London, United Kingdom; 3 Faculty Of Life Sciences And Medicine, King’s College London, London, United Kingdom; 4 Psychological Medicine, King’s College Hospital, London, United Kingdom

**Keywords:** intravenous, liaisonpsychiatry, acutedisturbance, agitation

## Abstract

**Introduction:**

The intravenous (IV) is one of the main parenteral routes for drug administration. Rapid onset of action, precise titration, patient-specific dosing and bypass of liver metabolism are a few of its advantages, while hypersensitivity reactions, adverse effects, infection risk and a higher overall cost some of its most debated downsides. Unlike other areas of Medicine, IV has been significantly under-utilized in Psychiatry.

**Objectives:**

This systematic review analyzed the evidence for effectiveness and safety behind the use of IV medication used for the management of acute disturbance.

**Methods:**

APA PsycINFO, MEDLINE, and EMBASE databases were searched for eligible studies. Studies were included if they used IV medication to treat acute disturbance, in English language, had participants aged >18. The quality of the included studies was assessed using the National Institutes of Health quality checklist.

**Results:**

17 studies were deemed eligible. Data analysis was limited to narrative synthesis since primary outcome measures varied significantly between each study. Findings showed strong evidence for efficacy and safety of dexmedetomidine, droperidol, midazolam, and olanzapine. These medications displayed a short time to sedation, reduction in agitation levels, or large percentage of patients adequately sedated with a low number of adverse events. Results did not provide enough evidence for the use of IV ketamine, haloperidol, diazepam, lorazepam, and promethazine.

**Conclusions:**

This review supports dexmedetomidine, droperidol, midazolam, and olanzapine as safe and efficacious options for managing acute disturbance via the intravenous route, particularly in special clinical settings where trained staff, optimal monitoring, resuscitation equipment and ventilators are all at hand.

